# Efficacy of liposomal amphotericin B and anidulafungin using an antifungal lock technique (ALT) for catheter-related *Candida albicans* and *Candida glabrata* infections in an experimental model

**DOI:** 10.1371/journal.pone.0212426

**Published:** 2019-02-19

**Authors:** Jana Basas, Marta Palau, Xavier Gomis, Benito Almirante, Joan Gavaldà

**Affiliations:** Antibiotic Resistance Laboratory, Infectious Diseases Department, Vall d’Hebron Research Institute (VHIR), Hospital Universitari Vall d’Hebron, Barcelona, Spain; Institute of Microbiology, SWITZERLAND

## Abstract

**Objective:**

The aims of this study were as follows. First, we sought to compare the *in vitro* susceptibility of liposomal amphotericin B (LAmB) and anidulafungin on *Candida albicans* and *Candida glabrata* biofilms growing on silicone discs. Second, we sought to compare the activity of LAmB versus anidulafungin for the treatment of experimental catheter-related *C*. *albicans* and *C*. *glabrata* infections with the antifungal lock technique in a rabbit model.

**Methods:**

Two *C*. *albicans* and two *C*. *glabrata* clinical strains were used. The minimum biofilm eradication concentration for 90% eradication (MBEC_90_) values were determined after 48h of treatment with LAmB and anidulafungin. Confocal microscopy was used to visualize the morphology and viability of yeasts growing in biofilms. Central venous catheters were inserted into New Zealand rabbits, which were inoculated of each strain of *C*. *albicans* and *C*. *glabrata*. Then, catheters were treated for 48h with saline or with antifungal lock technique using either LAmB (5mg/mL) or anidulafungin (3.33mg/mL).

**Results:**

*In vitro*: anidulafungin showed greater activity than LAmB against *C*. *albicans* and *C*. *glabrata* strains. For *C*. *albicans*: MBEC_90_ of anidulafungin versus LAmB: CA176, 0.03 vs. 128 mg/L; CA180, 0.5 vs. 64 mg/L. For *C*. *glabrata*: MBEC_90_ of anidulafungin versus LAmB: CG171, 0.5 vs. 64 mg/L; CG334, 2 vs. 32 mg/L. *In vivo*: for *C*. *albicans* species, LAmB and anidulafungin achieved significant reductions relative to growth control of log_10_ cfu recovered from the catheter tips (CA176: 3.6±0.3 log_10_ CFU, p≤0.0001; CA180: 3.8±0.1 log_10_ CFU, p≤0.01). For *C*. *glabrata*, anidulafungin lock therapy achieved significant reductions relative to the other treatments (CG171: 4.8 log_10_ CFU, p≤0.0001; CG334: 5.1 log_10_ CFU, p≤0.0001)

**Conclusions:**

For the *C*. *albicans* strains, both LAmB and anidulafungin may be promising antifungal lock technique for long-term catheter-related infections; however, anidulafungin showed significantly higher activity than LAmB against the *C*. *glabrata* strains.

## Introduction

*Candida* spp. are the fourth most common cause of nosocomial bloodstream infection (BSI) worldwide with an incidence of 8.1 cases/100 000 inhabitants in Spain during the years 2010–2011 and an attributable mortality rate of 31%.[1]

Although *Candida albicans* remains globally the most common fungal isolate from blood, substantial increases in the prevalence of non-*albicans Candida* spp. have been reported, with proportions of 45 and 55%, respectively.[1,2] Cases of non-*albicans Candida* BSI are commonly caused by *Candida parapsilosis* (24.9%), *Candida glabrata* (13.4%) and *Candida tropicalis* (7.7%).[1,2]

Central venous catheters (CVCs) appear to be the most common risk factor for the development of candidemia in patients without neutropenia or major immunodeficiencies. These infections are closely related to the ability of *Candida* to form biofilms on artificial surfaces.[3,4] This ability poses an important therapeutic issue because fungal cells encased in biofilms are known to display a reduced susceptibility to antifungals compared to their planktonic counterparts.[4] The treatment of long-term catheter-related BSI (CRBSI) caused by *Candida* spp. requires the removal of the catheter and in addition to systemic treatment with fluconazole or an echinocandin for two weeks,[5,6] although some studies have noted that the impact of this practice has failed to improve the outcome in subgroups of patients.[7] On the other hand, catheter removal is not always possible (i.e., in subsets of patients who have a surgically implantable catheter with no other available vascular access or who have profound thrombocytopenia); thus, the need arises for a therapeutic option that allows the conservative management of the catheter-related infection, such as the antifungal lock technique (ALT), which consists of locking catheters with a high concentration of an antifungal solution.[8] This conservative strategy would be useful in select circumstances, for example, in uncomplicated cases in which catheters are infected with *Candida* spp. but are greatly needed or unsafe to remove.

Few investigations published to date have been focused on the *in vivo* usefulness of ALT to treat *C*. *albicans* and *C*. *glabrata* catheter-related infections.[9] According to The Infectious Diseases Society of America (IDSA),[6] the data are insufficient to support specific recommendations for the use of ALT for catheter salvage in the management of CRBSI caused by *Candida* spp.

This study had two purposes. The first purpose was to assess the *in vitro* susceptibility of *C*. *albicans* and *C*. *glabrata* growing on silicone discs to LAmB and anidulafungin, and the second purpose was to assess the effectiveness of anidulafungin at 3.33 mg/mL compared with LAmB at 5 mg/mL for the treatment of experimentally induced *C*. *albicans* and *C*. *glabrata* catheter-related infections by the antifungal lock technique.

## Materials and methods

### Strains

Four well-characterized biofilm-producing *Candida* spp. strains were used. Two *C*. *albicans* (CA176 and CA180) and two *C*. *glabrata* (CG171 and CG334) strains were isolated from patients with CRBSI. For quality control, the ATCC *C*. *parapsilosis* 22019 reference strain was used. The strains were stored at -80°C in skim milk.

### Antifungals

Liposomal amphotericin B (LAmB) was provided by Gilead Sciences, Inc. (Madrid, Spain), and anidulafungin was provided by Pfizer, Inc. (Madrid, Spain). For the *in vivo* animal model of infection, the concentrations used were 5 mg/mL for LAmB and 3.33 mg/mL for anidulafungin. These concentrations were the highest possible due to the concentration provided in the commercial drug vial. The drug dilutions were prepared according to the manufacturers’ instructions. The treatments were supplemented with 100 IU/mL of sodium heparin.

### *In vitro* studies

#### Susceptibility studies

The MICs of LAmB and anidulafungin for the two *C*. *albicans* and the two *C*. *glabrata* strains were determined by the broth microdilution method according to the European Committee on Antimicrobial Susceptibility Testing (EUCAST) guidelines and breakpoints.[10] For the biofilm formation assay, we followed the protocol described by Chandra *et al*.,[11] with slight modifications. The *C*. *albicans* and *C*. *glabrata* strains were grown overnight in Yeast Nitrogen Base medium (Sigma-Aldrich Co., Madrid, Spain) with 50 mM dextrose (YNBD) at 37°C with continuous shaking at 60 rpm. After centrifugation, the cultures were washed twice with sterile PBS (pH 7.2) and were resuspended at a final concentration of 1·10^7^ blastoconidia/mL. Then, inocula were applied to 12-well plates, and silicone elastomer discs (15 mm diameter x 1.5 mm; Merfasa SL, Barcelona, Spain) were added. The silicone discs were incubated for 90 min at 37°C and were then placed in a new plate containing brain heart infusion (BHI; Becton, Dickinson and Company, Le Pont de Claix, France) medium. These plates were incubated for 48 h at 37°C with continuous shaking at 60 rpm. The silicone discs that contained the fungal biofilm were placed on a new plate containing various two-fold dilutions of LAmB and anidulafungin and were incubated at 37°C for 48 h. For the evaluation of the minimum biofilm eradication concentration for 90% eradication (MBEC_90_), the silicone discs were placed in a new plate with fresh BHI. The biofilm was scraped (Sarstedt, Inc., Newton, NC, USA) and serially diluted, and the yeast cells were enumerated by the viable count method. The MBEC_90_ was defined as the minimum concentration of antimicrobial required to reduce the biofilm cell numbers (relative to the initial inoculum size) by ≥90%.[12] All experiments were performed in triplicate.

#### Confocal laser scanning microscopy assay

The effect of different concentrations of LAmB and anidulafungin on the growth of *C*. *albicans* and *C*. *glabrata* biofilm on silicone discs was visualized using confocal laser scanning microscopy (CLSM) with an Olympus FV1000 microscope with excitation wavelengths of 488 and 568 nm. To grow the biofilms on the silicone discs, yeast cells were cultured in BHI at 37°C while being shaken for 48 h, as described previously.[11] Then, biofilms were treated for 48 h with LAmB or anidulafungin at 37°C. Afterwards, the biofilms were stained using a LIVE/DEAD BacLight Viability Kit (Molecular Probes, Invitrogen, Leiden, The Netherlands). Briefly, this technique consisted of staining the cells encased in the biofilm with a mixture of SYTO 9 (3.34 mM solution in DMSO) and propidium iodide (20 mM solution in DMSO) and incubating at room temperature in the dark for 30 min to distinguish between dead cells and cells that remained alive after antifungal exposure. Three areas of the biofilm on each silicone disc were scanned with a 2 μm step size. Simultaneous dual-channel imaging was used to display the green and red fluorescence. The IMARIS 8 software (Bitplane, Belfast, UK) was used to create a projection view of the formed biofilms, and the ImageJ 1.45s software package (developed at the National Institutes of Health, Bethesda, USA) was used to calculate the percentage of green (live) and red (dead) pixels. All experiments were performed in triplicate.

### *In vivo* studies

#### Ethics statement

This study was carried out in strict accordance with the recommendations in the Guide for the Care and Use of Laboratory Animals of the National Institutes of Health and the ARRIVE Guidelines (Animal Research: Reporting of *In vivo* Experiments) (S1 and S2 files), and all efforts were made to minimize suffering. The experimental protocol was approved by the Animal Experimentation Ethics Committee of Vall d’Hebron Research Institute (registration number 73.12 CEEA) and the Ministry of Environment of the Catalan Government (registration number 7863).

#### Catheter-related infection model

The animal model was based on our previous studies.[12] New Zealand white male rabbits (Granja Riera, Barcelona, Spain) weighing 2.0 to 2.2 kg were singly housed under a reversed 12 h/12 h light/dark cycle with water and food *ad libitum* throughout the experiment. Briefly, animals were anaesthetized by intramuscular injection of 100 mg/kg ketamine plus 20 mg/kg xylazine. Then, a catheter made of sterile silicone tubing (SILASTIC, ID/OD: 0.45/0.77 inch, Dow Corning Corporation, MI, USA) was inserted into the internal jugular vein at a depth of up to 8 cm and secured with silk suture. After placement, the catheters were inoculated with 0.35 mL of a suspension containing 1·10^7^ blastoconidia/mL of either *C*. *albicans* or *C*. *glabrata* strains. The inoculum was locked in the lumen of the catheter for 48 h and was carefully withdrawn by aspiration immediately before starting ALT. The treatment groups were as follows: untreated control (normal saline), LAmB at 5 mg/mL and anidulafungin at 3.33 mg/mL. The catheters were locked with 0.4 mL of each treatment for 48 h. At the end of the treatment period, the animals were euthanized with intravenous pentobarbital, and the catheters were removed for microbiological evaluation. The distal 4 cm of each catheter was cut, submerged in BHI and sonicated at 50 Hz for 10 min for quantitative culture. The sonication products were washed twice by centrifugation, and the pellet was serially diluted, plated, and incubated on Sabouraud agar (Sigma-Aldrich Co., Madrid, Spain) plates for 48 h at 37°C. After incubation, a colony count was performed. The results were expressed as log_10_ total colony-forming units (CFU).

#### Statistical analysis

The catheters yielding negative results were compared. The percentage of negative cultures obtained in each treatment was analysed using Fisher’s exact test, and the mean of log_10_ CFU recovered from the catheter tips was compared using one-way ANOVA and Tukey’s *post-hoc* test. Statistical analysis was performed using the Statistical Package for the Social Sciences (SPSS, Inc., Chicago, IL, USA) version 16 package program. P-values of ≤ 0.05 were considered statistically significant. P-values > 0.05 were not statistically significant (p NS).

## Results

### *In vitro* studies

#### Susceptibility studies

The results of the susceptibility study can be seen in Table 1; the *C*. *albicans* and *C*. *glabrata* isolates used in our study were susceptible to both LAmB and anidulafungin, according to the interpretation of susceptibility defined by the most recent EUCAST breakpoints.[10]

**Table 1:** *In vitro* susceptibility of *Candida albicans* and *Candida glabrata* strains to LAmB and anidulafungin.

		LAmB		Anidulafungin	
	Strain	MIC (mg/L)	MBEC_90_ (mg/L)	MIC (mg/L)	MBEC_90_ (mg/L)
***C*. *albicans***	**CA176**	0.5	128	0.0017	0.03
	**CA180**	0.5	64	0.0035	0.5
***C*. *glabrata***	**CG171**	1	64	0.015	0.5
	**CG334**	0.5	32	0.007	2

LAmB, Liposomal Amphotericin B; MIC, minimum inhibitory concentration; MBEC_90_, minimum biofilm eradication concentration for 90% eradication.

Anidulafungin was >1000-fold more effective than LAmB against both *C*. *albicans* strains growing on silicone discs (MBEC_90_ anidulafungin vs. MBEC_90_ LAmB: CA176, 0.03 vs. 128 mg/L; CA180, 0.5 vs. 64 mg/L).

For the *C*. *glabrata* strains growing on silicone discs, anidulafungin was >10 and >100-fold more effective than LAmB against the CG171 and CG334 strains, respectively (MBEC_90_ anidulafungin vs. MBEC_90_ LAmB: CG171, 0.5 vs. 64 mg/L; CG334, 2 vs. 32 mg/L).

#### Confocal laser scanning microscopy assay

**Fig 1:**
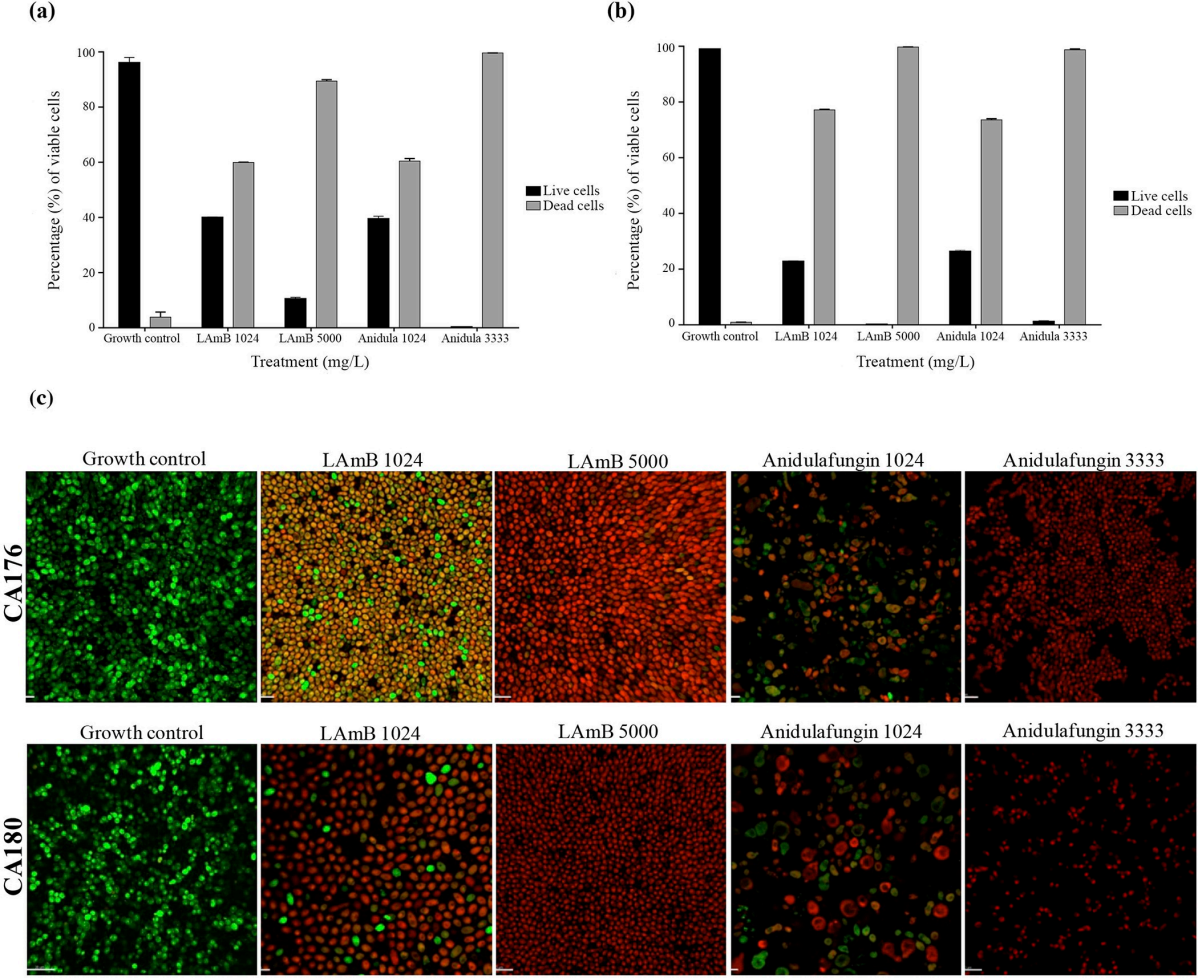
Viability assay for biofilms of *Candida albicans* strains. (A) Viability of strain CA176. (B) Viability of strain CA180. (C) LIVE/DEAD fluorescence imaging of strains CA176 and CA180. LAmB, Liposomal Amphotericin B; AFG, anidulafungin.

**Fig 2:**
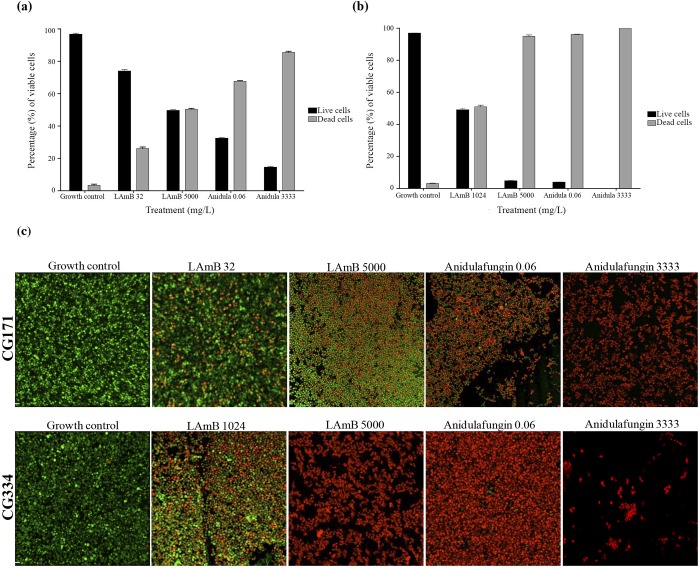
Viability assay for biofilms of *Candida glabrata* strains. (A) Viability of strain CG171. (B) Viability of strain CG334. (C) LIVE/DEAD fluorescence imaging of strains CG171 and CG334. LAmB, Liposomal Amphotericin B; AFG, anidulafungin.

CLSM was used to visualize the morphology and viability of *Candida* spp. growing on silicone discs for 48 h using a LIVE/DEAD staining technique. Figs 1(A), 1(B), 2(A) and 2(B) represent the percentage of viable cells after 48 h of treatment against the tested strains of *C*. *albicans* and *C*. *glabrata*, respectively.

At high concentrations, LAmB and anidulafungin had similar effects on the reduction of cell viability in the biofilms of both *C*. *albicans* strains. The reduction in cell viability due to treatment with anidulafungin at 3333 mg/L approached or exceeded 90% for both the CA176 (Fig 1A) and CA180 (Fig 1B) strains; LAmB at 5000 mg/L had a similar effect.

Regarding the *C*. *glabrata* strains, the effects of high concentrations of LAmB and anidulafungin were different for the two strains studied. Anidulafungin at 3333 mg/L demonstrated better efficacy (87% cell death) than LAmB at 5000 mg/L against the CG171 strain (Fig 2A). In contrast, LAmB and anidulafungin had a similar effect (cell death greater than 90%) against the CG334 strain (Fig 2B). Note that in response to anidulafungin at a low concentration of 0.06 mg/L, the reduction in *C*. *glabrata* viability was greater than 70% for both strains (CG171: 70%; CG334: 96%).

The visualization of *Candida* spp. morphology by CLSM (Figs 1C and 2C: 60x magnification) revealed small, ovoid colonies in the groups treated with different concentrations of LAmB and high concentrations of anidulafungin; this morphology was the same as that observed in the growth control group. Interestingly, we observed a large, globose morphology in the *C*. *albicans* strains when biofilms of these strains were treated with low concentrations of anidulafungin, suggesting a structural defect in the cell surface of these strains. However, this effect was not observed in the *C*. *glabrata* strains.

### *In vivo* studies

#### Catheter-related infection model

The results obtained from the rabbit catheter tip cultures after sonication and centrifugation are shown in Table 2. For catheter-related infection due to either strain of *C*. *albicans*, the fungal load recovered from the catheter tips in both the anidulafungin- or LAmB-lock therapies treated animals and the untreated control animals was similar (CA176: 3.6 ± 0.3 log_10_ CFU, p ≤ 0.0001; CA180: 3.8 ± 0.1 log_10_ CFU, p ≤ 0.01). However, there were differences between the two strains with respect to the negativization of the catheter tips. In the rabbits infected with the CA176 strain, only half of the catheters in the LAmB lock therapy group were sterile (control group: 0%; anidulafungin group: 40%; p ≤ 0.05 vs. untreated control group). In the animals infected with the CA180 strain, the anidulafungin- and LAmB-lock therapies achieved the same rate of catheter tip negativization as did no treatment (83% in both treatments, p ≤ 0.01).

**Table 2:** Results of antifungal lock therapy for *Candida albicans* and *Candida glabrata* strains.

Treatment	*C*. *albicans*				*C*. *glabrata*			
	CA176		CA180		CG171		CG334	
	Negative / total (%)	Log_10_ CFU mean ± SD (95% CI)	Negative / total (%)	Log_10_ CFU mean ± SD (95% CI)	Negative / total (%)	Log_10_ CFU mean ± SD (95% CI)	Negative / total (%)	Log_10_ CFU mean ± SD (95% CI)
**Control**	0/10 (0)	5.1 ± 0.5 (4.8–5.5)	0/4 (0)	4.0 ± 1.0 (2.5–5.5)	0/10 (0)	5.7 ± 0.4 (5.5–6.1)	0/7 (0)	5.1 ± 0.7 (4.4–5.8)
**LAmB 5 mg/mL**	5/10 (50) ^a^	1.2 ± 1.3 (0.2–2.0) ^b^	5/6 (83) ^c^	0.3 ± 0.8 (0.5–1.2) ^c^	3/14 (21)	2.1 ± 1.2 (1.4–2.8) ^b^	2/7 (29)	1.4 ± 1.0 (0.4–2.3) ^b^
**AFG 3.33 mg/mL**	4/10 (40)	1.8 ± 1.5 (0.7–2.9) ^b^	5/6 (83) ^c^	0.2 ± 0.7 (0.4–1.0) ^c^	7/11 (64) ^c^ ^d^	0.9 ± 1.3 (0.1–1.8) ^b^ ^d^	8/8 (100) ^b^ ^e^	0.0 ± 0.0 (0.0–0.0) ^b^ ^e^

The results are given as the ratio of the negative catheter cultures to the total number of catheters (expressed as percentages), and the median total CFU (expressed in log_10_ CFU). LAmB, Liposomal amphotericin B; AFG, Anidulafungin.

^a^p *≤* 0.05 vs. control

^b^p *≤* 0.0001 vs. control

^c^p *≤* 0.01 vs. control.

^d^p *≤* 0.05 vs. LAmB.

^e^p*≤* 0.01 vs. LAmB.

In the animals infected with either strain of *C*. *glabrata*, the colony counts from the catheter tips after 48 h of antifungal lock therapy were significantly lower in the animals treated with lock therapy with anidulafungin than in those treated with lock therapy with LAmB (CG171, p ≤ 0.05; CG334, p ≤ 0.01). Compared with no treatment, lock therapy with anidulafungin at 3.33 mg/mL resulted in a significant reduction in CFU recovered from the catheter tips; this reduction was more than 4.8 log_10_ CFU for both strains of *C*. *glabrata* (CG171: 4.8 log_10_ CFU, p ≤ 0.0001; CG334: 5.1 log_10_ CFU, p ≤ 0.0001). Note that anidulafungin at 3.33 mg/mL was the only antifungal treatment that resulted in a significant percentage of negative catheter cultures for both strains after 48 h: 64% for strain CG171 (control: 0%; LAmB 5 mg/mL: 21%; p ≤ 0.05) and 100% for strain CG334 (control: 0%; LAmB 5 mg/mL: 29%; p ≤ 0.01).

## Discussion

Focusing on the *in vitro* MBEC_90_ assays, we found that anidulafungin was more effective than LAmB against *C*. *albicans* and *C*. *glabrata* biofilms growing on silicone discs. In contrast, we obtained different results in the cell viability tests. Both anidulafungin at 3.33 mg/mL and LAmB at 5 mg/mL had a similar efficacy (cell death >90%) against *C*. *albicans* strains. In contrast, against *C*. *glabrata*, anidulafungin demonstrated better efficacy than LAmB against the CG171 strain, and both treatments (anidulafungin and LAmB) achieved a similar effect against the CG334 strain (cell death >90%).

Rapid salvage after catheter-related infection is likely to be clinically important because it allows earlier access to long-term catheters, thereby improving patient outcomes. This rapid salvage effect was obtained by lock therapy with either 3.33 mg/mL of anidulafungin or 5 mg/mL of LAmB for 48 h in our experimental model of *C*. *albicans* catheter-related infection. Note that although both antifungals decreased the fungal load in the catheters infected with the two strains of *C*. *albicans*, a significant sterilization of the catheter tips was only achieved for one of the two strains studied. In contrast, in the experimental model of *C*. *glabrata* catheter-related infection, lock therapy with anidulafungin resulted in a significant reduction in fungal load recovered from the catheter tips compared to that achieved by LAmB for both strains. Likewise, only anidulafungin treatment resulted in negative catheter tip cultures. These data are in accordance with our previous published study, in which lock therapy with anidulafungin showed the highest activity against experimental catheter-related *C*. *parapsilosis* infection.[12] Our study thus identifies ALT as a fast and highly effective treatment that is an alternative to catheter removal for those selected cases; however, the ALT regimen assessed in our study is *Candida* species-dependent.

Our results are similar to those reported in an experimental model of *C*. *albicans* infection, which demonstrated no difference between ALT with caspofungin or LAmB;[13–15] however, Schinabeck *et al*.,[13] Chandra *et al*.,[14] and Shuford *et al*.[15] demonstrated catheter sterilization after 7 days of ALT with LAmB, deoxycholate AmB (dAmB), or caspofungin. Our studies only treat with ALT for 48 h because we believe that rapid clearance of the pathogen in an infected central venous catheter would likely be clinically important. In contrast to earlier findings, which argue that there were no differences between ALT with caspofungin or LAmB against *C*. *glabrata* isolates, our model of ALT demonstrates paradoxical behaviour of *C*. *glabrata* biofilms on the catheters in response to LAmB and anidulafungin.

In general, the published *in vitro* data have shown that dAmB and echinocandins such as caspofungin or micafungin have high activity against *C*. *albicans* and *C*. *glabrata* growing in biofilms.[16] Nevertheless, anidulafungin and lipid formulations of AmB have been rarely investigated using *in vitro* susceptibility biofilm models. Oncu *et al*.[17] tested the effect of dAmB compared with caspofungin against *C*. *albicans* on silicone catheter segments and demonstrated complete growth inhibition. Ramage *et al*.[18] and Kawai *et al*.[19] demonstrated a dose-dependent activity of LAmB against *C*. *albicans* and *C*. *glabrata* biofilms, a result similar to that observed in our study. Also in accordance with our findings, Cateau *et al*.[20] demonstrated the efficacy of 48 h of echinocandin (caspofungin and micafungin) treatment against young and mature biofilms of *C*. *albicans* and *C*. *glabrata* on silicone catheter segments.

Our results show that after exposing biofilms of *C*. *albicans* and *C*. *glabrata* growing on silicone discs to different concentrations of LAmB and anidulafungin, the MBEC_90_ antifungal concentration of obtained after the discs were scraped and cultivated in Sabouraud agar was lower than the viability observed with the LIVE/DEAD technique and visualized with CLSM. This finding is in agreement with the results of Salma *et al*.,[21] which showed the existence of a viable but nonculturable (VBNC) state in *S*. *cerevisiae*. The concept of a VNBC state has been thoroughly studied in bacteria but not in yeast, and our study is the first to describe such a state in *Candida* spp. This phenomenon is characterized by the ability of the cells to maintain detectable metabolic activity but an inability to grow on culture media, and it arises as the result of the stress response; in this case, the stress is a result of exposure to antifungals. The recovery of cultivability is possible if the stress is removed, but only if the cells were in this state for a short period of time.

Few data have been published regarding the conservative management of CRBSI caused by *Candida* spp. in humans. Imbert *et al*.[16] have recently reviewed the literature discussing the antifungal solutions used for lock therapy and have reported all of the clinical studies with *C*. *albicans* and *C*. *glabrata*. Regarding cases of catheter-related *C*. *albicans* infection; 5 out of 5 patients treated with ALT using dAmB at 2 to 2.5 mg/mL demonstrated catheter salvage,[22–24] and Buckler *et al*.[25] achieved a catheter salvage rate of 50% (1 out of 2 cases) using lock therapy with LAmB at 2.67 mg/mL. Four cases of catheter-related *C*. *glabrata* infection using lock therapy with AmB have been reported;[23] only 1 out 3 cases achieved catheter salvage using dAmB at 2.5 mg/mL, and one patient was treated and cured by using ALT with dAmB at 5 mg/mL. Interestingly, the literature does not report the use of anidulafungin-lock therapy to treat catheter-related *C*. *albicans* or *C*. *glabrata* infection.

In conclusion, ALT with anidulafungin provided a fast decrease in biofilm-embedded concentrations of *C*. *albicans* and *C*. *glabrata* and resulted in negative catheter tip cultures after 48 h of treatment in the majority of animals. For catheter-related *C*. *albicans* infection, ALT with either anidulafungin or Liposomal Amphotericin B was equally effective in eradicating infection, although one of the two tested strains did not attain negative catheter cultures at 48 h. Nevertheless, against *C*. *glabrata*, only ALT with anidulafungin at 3.33 mg/mL resulted in a high percentage of negative catheter tip cultures at the critical time point of 48 h; thus, this regimen may be useful as an antifungal lock therapy in the treatment of catheter-related infection due to *C*. *glabrata*. The results of this study support lock therapy with anidulafungin at 3.33 mg/L or Liposomal Amphotericin B at 5 mg/L as an adjunctive therapeutic option to systemic treatment in selected cases of long-term CRBSI caused by *C*. *albicans*. In patients with long-term CRBSI caused by *C*. *glabrata*, we recommend anidulafungin as an elective antifungal lock therapy.

## Supporting information

S1 File ARRIVE Guidelines checklist. (PDF)

S2 File ARRIVE Guidelines checklist attachment. (PDF)
