# Supplementary material for: Efficacy of liposomal amphotericin B and anidulafungin using an antifungal lock technique (ALT) for catheter-related Candida albicans and Candida glabrata infections in an experimental model
Source: PLoS One. 2019 Feb 19;14(2):e0212426. doi: 10.1371/journal.pone.0212426 (PMC6380555; doi:10.1371/journal.pone.0212426)
Supplement: S2 File — (PDF) [file pone.0212426.s002.pdf]

Attachment to The ARRIVE Guidelines Checklist:

Item 1: Please see title section of the manuscript (lines 1 to 3).

Item 2: Please see abstract section of the manuscript (lines 27 to 53).

Item 3: a) Please see Introduction section of the manuscript (lines 56 to 90). **b)** The rabbit model of catheter-related infection is widespread. The smallest gauge for the internal jugular vein (access route) makes rabbits the chosen animal for this model, allowing an easy surgery for the placement of the catheter and also makes possible to obtain enough sample volume to perform further microbiological studies.

Item 4: Please see Introduction section of the manuscript (lines 86 to 90).

Item 5: Please see Materials and Methods (Ethics) section of the manuscript (lines 150 to 157). The experimental protocol performed in strict accordance with the recommendations in the *Guide for the Care and Use of Laboratory Animals* and the ARRIVE Guidelines (Animal Research: Reporting of *In vivo* Experiments), and all efforts were made to minimize suffering. All the experimental procedures were performed in accordance with the Catalan (Decree 214/1997 30 July. Use of Animals for experimentation and other scientific purposes. Regulation. DOCG 2450; 7/8/1997), Spanish (Royal Decree 53/2013, of February 1, by which the basic standards are established for the protection of animals used for experimental and other scientific purposes, including education) and European (European Directive 2010/63/EU on the protection of animals used for scientific purposes) laws and regulations on the protection of animals used for experimental and other scientific purposes, so we verify that the institution meets all bioethical requirements. Moreover, the experimental protocol was approved by the Animal Experimentation Ethics Committee of Vall d'Hebron Research Institute (registration

number 73/12 CEEA) and the Ministry of Environment of the Catalan Government (registration number 7863).

Item 6: a) Please see Materials and Methods section of the manuscript (lines 169 to 171). **b)** Animals were randomly selected. We marked each animal and each group of treatment to permit individual identification and kept in their cages for at least 7 days before the experimental to allow for acclimatization to the experimental conditions. **c)** Please see Materials and Methods section of the manuscript (lines 162 to 164).

<u>Time-line diagram:</u>

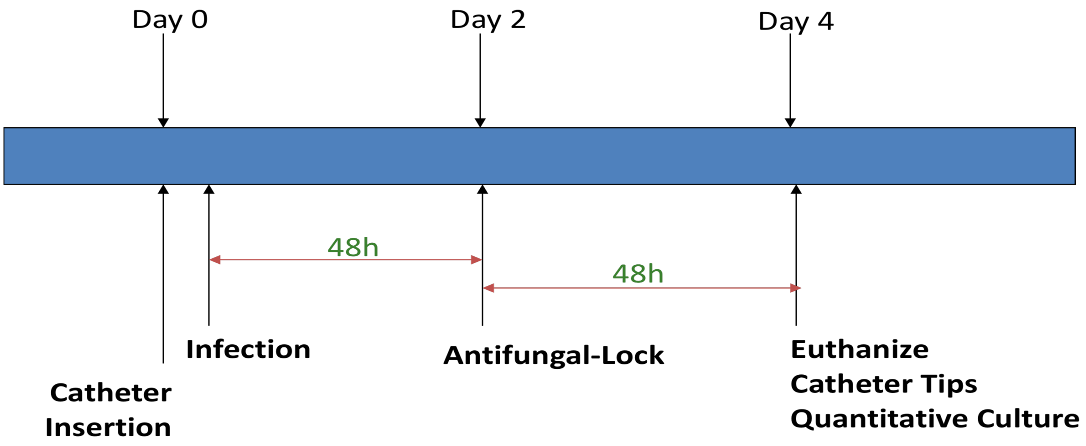

Item 7: a-c) Please see Materials and Methods (Antifungals) section of the manuscript (lines 100 to 106) for drug formulation and dose, please see materials and Methods section of the manuscript (lines 159 to 178) for anaesthesia used, surgical procedure, method of euthanasia, etc. **d)** All care was taken to cause no pain to the animals. For surgery, we used anaesthesia with ketamine and xylazine. Ketamine is a short-acting anaesthetic with an induction time of half an hour during which we can inserted catheter in the internal jugular vein. Xylazine has proven to be a safe anaesthetic adjunct when co administered with ketamine to induce short periods of surgical anaesthesia. When combined with ketamine, muscle relaxation and visceral analgesia are improved, and emergence from anaesthesia is smoother. Intramuscular is the preferred route of administration of this anaesthetic.

Item 8: a-b) Please see Materials and Methods section of the manuscript (lines 155 to 157).

Item 9: Please see Materials and Methods section of the manuscript (lines 160 to 162). **a)** The housing: animals were singly housed to avoid catheter removal among rabbits **b)** The husbandry conditions: -Light/dark cycle- 12 h/12 h. Animal Room Temp: 22 +/- 2ºC, relative humidity: 30-70%, access to feed and water: *ad libitum.* **c)** Animals were kept in their cages for at least 7 days before the experiment to allow for acclimatization to the experimental conditions. After experimental surgery, animals were closely monitored daily during all the experiment. During experimental, no onset of abnormality was found.

Item 10: a) We used 103 animals for all the experiments. In the catheter-related infection by *C. albicans* CA176 we used 30 animals; 10 animals in control group, 10 animals in Liposomal Amphotericin B (LAmB) 5 mg/mL and 10 animals in anidulafungin 3.33 mg/mL. In the catheter-related infection by *C. albicans* CA180 we used 16 animals; 4 animals in control group, 6 animals in Liposomal Amphotericin B (LAmB) 5 mg/mL and 6 animals in anidulafungin 3.33 mg/mL.

In the catheter-related infection by *C. glabrata* CG171 we used 35 animals; 10 animals in control group, 14 animals in Liposomal Amphotericin B (LAmB) 5 mg/mL and 11 animals in anidulafungin 3.33 mg/mL. In the catheter-related infection by *C. glabrata* CG334 we used 22 animals; 7 animals in control group, 7 animals in Liposomal Amphotericin B (LAmB) 5 mg/mL and 8 animals in anidulafungin 3.33 mg/mL. **b)** Fewer animals were used in some experimental groups given that the size of the group was broad enough to detect a difference of 1.5 log colony-forming units/g with an α error of 0.05 and a β error of 0.20. To observe significant differences between groups 5 animals/group were necessary. In order to arrive at this number of animals we used Statistical program GRANMO 5.2. **c)** We duplicate the experiment to ensure reproducibility.

Item 11: a-b) Animals were randomly selected. We marked each animal and each group of treatment to permit individual identification. We separate the experiments for the yeast strains. Firstly we did the catheter-related infection with *C. albicans* strains and then we did the model with *C. glabrata* strains.

Item 12: Please see materials and Methods section of the manuscript (lines 173 to 178).

Item 13: a-c) Please see Materials and Methods (Statistical analysis) section of the manuscript (lines 180 to 186). For the statistical analysis we used SPSS 16.0 software. Fewer animals were used in some experimental groups given that the size of the group was broad enough to detect a difference of 1.5 log CFU/g with an α error of 0.05 and a β error of 0.20. To observe significant differences between groups 5 animals/group were necessary; in order to arrive at this number of animals we used Statistical program GRANMO 5.2. We duplicate the experiment to ensure reproducibility.

Item 14: All the rabbits in the experimental groups were of the weight of 2.0 to 2.2 kg prior to treatment or testing. These animals had not been previously used for any procedure.

Item 15: a) Please see Table 2 section of the manuscript (lines 258 to 259). **b)** All the animals with *Candida* spp. catheter-related infection survived the entire experimental period; moreover, some rabbits were excluded because they removed the catheters before the end of the study. We include all the minimum of necessary animals in each group (5 animals/group) to observe significant differences between groups. We duplicate the experiment to ensure reproducibility.

Item 16: a) Please see Results (*In vivo* studies) section of the manuscript (lines 238 to 258) and Table 2 section of the manuscript.

Item 17: a) In our model of catheter-related infection of *Candida* spp., we did not have important adverse events in each experimental group **b)** Being that we used this model in other experimental procedures (registration number 73/12 CEEA), we did not any modifications due to we saw none of adverse events in the previous studies.

Item 18: a Please see Discussion section of the manuscript (lines 261 to 343). The major limitation of catheter-related infection studies are the impossibility of maintaining ALT during 14 days (as in humans). For this reason, we used an animal model of 48 h that allows us to see the efficacy of the treatment and the eradication of the biofilm. **c)** As described in our manuscript, the use of silicone discs for the *in vitro* model of ALT will be a reduction for studying ALT whereas with this substrate, we attached the same results as *in vivo* model of ALT.

Item 19: Please see Discussion section of the manuscript (lines 261 to 343).

Item 20: This study was supported by research grants from the Ministerio de Sanidad y Consumo, Instituto de Salud Carlos III (FIS PI070394) and was co-financed by the European Development Regional Fund "A way to achieve Europe" ERDF, the Spanish Network for Research in Infectious Diseases (REIPI RD12/0015), and partially by Pfizer, Inc. The funders had no involvement in the design and conduct of the study; analysis, and interpretation of the data; and preparation, review, or approval of the manuscript; and decision to submit the manuscript for publication.
